# The Plant Defensin NaD1 Enters the Cytoplasm of *Candida albicans* via Endocytosis

**DOI:** 10.3390/jof4010020

**Published:** 2018-02-06

**Authors:** Brigitte M. E. Hayes, Mark R. Bleackley, Marilyn A. Anderson, Nicole L. van der Weerden

**Affiliations:** La Trobe Institute for Molecular Science, La Trobe University, 3086 Melbourne, Australia; B.Hayes@latrobe.edu.au (B.M.E.H.); m.bleackley@latrobe.edu.au (M.R.B.); M.Anderson@latrobe.edu.au (M.A.A.)

**Keywords:** fungi, antifungal peptides, plant defensin, endocytosis, *Candida albicans*

## Abstract

Antimicrobial peptides are widespread in nature and are produced by many organisms as a first line of defence against pathogens. These peptides have a broad range of biological activities, such as antibacterial or antifungal activities and act with varied mechanisms of action. A large number of the peptides are amphipathic α-helices which act by disrupting plasma membranes and allowing leakage of intracellular contents. However, some peptides have more complex mechanisms of action that require internalisation into the target organisms’ cytoplasm. The method by which these peptides enter the cytoplasm varies, with some requiring the energy dependent processes of endocytosis or polyamine transport and others entering via passive transport. Here we describe the mechanism that the antimicrobial peptide, the plant defensin NaD1, uses to transverse the fungal membrane and gain access to the fungal cytoplasm. By inhibiting ATP synthesis and using an inhibitor of actin polymerisation, we show that NaD1 is internalised into *C. albicans* yeast cells by the energy-dependent process of endocytosis.

## 1. Introduction

Fungal disease is an increasing problem affecting both agriculture and human health [1,2]. With increasing incidence of human disease, particularly in immunocompromised individuals and the emergence of resistance to the current antifungal therapies [3,4,5,6], antimicrobial peptides are an attractive alternative method of treatment [7]. Antimicrobial peptides are innate immunity molecules produced by all organisms including plants, animals and insects [8]. Antimicrobial peptides are typically small, cationic molecules and many form amphipathic α-helices which act by binding to and disrupting lipid bilayers [8,9]. However, there are also more complex antifungal peptides that do more than simply disrupting membranes [10].

One such group of peptides is the plant defensins, many of which are potent antifungal molecules [11]. We have previously described the plant defensin NaD1, from ornamental tobacco, which has potent activity against both agricultural and human pathogens [12,13]. The mechanism of action of NaD1 is complex. Firstly, it interacts with components of the fungal cell wall [14]. The peptide then enters the cytoplasm and reactive oxygen species are produced [12,13]. The plasma membrane is then permeabilised and cell death occurs. Similarly, another plant defensin MtDef4 from the model legume *Medicago tranculata* enters the *Fusarium graminearum* cytoplasm before membrane permeabilisation and cell death [15]. We have hypothesised that permeabilisation of the membrane after NaD1 treatment is a result of interaction with phosphatidylinositol 4,5 bisphosphate (PI(4,5)P_2_) on the inner leaflet of the membrane. NaD1 binds to PI(4,5)P_2_ on lipid strips and can permeabilise PI(4,5)P_2_ containing liposomes [16]. However, the mechanism by which NaD1 gains access to the cytoplasm has not been elucidated.

Different mechanisms have been described for passage of antimicrobial peptides through the plasma membrane, including endocytosis, polyamine transporters and passive transport. For example, the *Penicillium* antimicrobial peptide PAF is internalised by endocytosis. This was revealed when PAF did not enter cells that had been treated with carbonyl cyanide m-chlorophenyl hydrazone (CCCP), an uncoupler of oxidative phosphorylation [17], or cells maintained at 4 °C indicating that energy is required for PAF uptake [18]. In addition, inhibition of actin polymerisation, which is required for endocytosis in yeast [19,20] blocks PAF internalisation into *Aspergillus nidulans* hyphae [18]. Similarly, uptake of the plant defensin MtDef4 into *Neurospora crassa* hyphae is reduced at 4 °C and was abolished when ATP production was blocked with sodium azide. Uptake of this defensin was also reduced in *N. crassa* cells after treatment with brefeldin A, which blocks retrograde transport and filipin, an inhibitor of lipid raft dependent endocytosis [15].

Polyamine transporters also function in the uptake of cationic peptides. For example, the human antifungal peptide, histatin 5, enters *C. albicans* cells via the polyamine transporters Dur3p and Dur31p. Deletion of these transporters reduces histatin 5’s antifungal activity [21] and expression of the transporters in a histatin 5 resistant *Candida glabrata* strain renders them sensitive to the peptide [22]. In addition, when the polyamines spermidine or spermine were added to *C. albicans* along with histatin 5, cells were more resistant to the peptide. Furthermore, uptake of FITC labelled histatin 5 into *C. albicans* was blocked by addition of spermidine [21]. Like PAF, CCCP, the uncoupler of oxidative phosphorylation, also blocks the antifungal activity of histatin 5, although in this instance it is likely due to impairment of the energy requirement of the polyamine transporter, as CCCP also blocks the uptake of spermidine into *C. albicans* cells [21].

Passive transport is the mode of entry of certain cell-penetrating peptides (CPPs) in accessing the cytoplasm. These peptides are small (less than 30 amino acids long) and positively charged [23]. One such CPP is the synthetic peptide transportan, a 27 residue variant of the peptide galparan, which was derived by fusion of the neuropeptide, galanin, with the wasp venom peptide, mastoparan [24]. Passage of this peptide across the plasma membrane and entry into the cytoplasm is likely to occur via direct penetration, as uptake is not inhibited by low temperatures (4 °C), nor is it blocked by phenylarsine oxide, an inhibitor of clathrin-mediated endocytosis, phagocytosis and macropinocytosis [20,23,24].

In this study, we investigated the mechanism by which the plant defensin NaD1 enters the cytoplasm of *C. albicans* cells. We show that NaD1 uptake is essential for killing and that uptake occurs through the energy dependent process of endocytosis. Furthermore, we show that a secondary method of killing that does not require endocytosis may occur at higher NaD1 concentrations and that once internalised, NaD1 does not require the cell’s internal protein transport machinery to have antifungal activity.

## 2. Materials and Methods

### 2.1. Protein Source

NaD1 was purified from the flowers of *Nicotiana alata* as described in van der Weerden et al. (2008) [12]. In brief, flowers were crushed in a mortar and pestle with liquid nitrogen and then subjected to an acid and heat treatment. Protein was then purified using cation-exchange chromatography and reverse-phase high-performance liquid chromatography (RP-HPLC). The protein concentration was determined using the bicinchoninic acid (BCA) protein assay (ThermoFisher, Scoresby, Australia). NaD1 was fluorescently labelled with 4,4-Difluoro-5,7-Dimethyl-4-Bora-3a,4a-Diaza-s-Indacene-3-Propionyl Ethylenediamine, Hydrochloride (BODIPY-FL-EDA, Life Technologies, Carlsbad, CA, USA) as described in [13]. The LL-37 peptide (amino acid sequence: LLGDFFRKSKEKIGKEFKRIVQRIKDFLRNLVPRTES) was synthesised by GenScript (Piscataway, NJ, USA).

### 2.2. Fungal Strains

All *C. albicans* strains were obtained from the fungal genetic stock centre [25] and are described in [26,27,28]. Strains were derived from the BWP17 background strain (*ura3*::*imm434/ura3*::*imm434 iro1/iro1*::*imm434 his1*::*hisG/his1*::*hisG arg4/arg4*). The DAY185 wild-type *C. albicans* strain (*ARG4*+, *URA3*+, *HIS1+*) was used in most assays. For testing of *C. albicans* ESCRT mutants, DAY286 (*ARG4*+, *URA3*+, *his1-*) was used as the wild-type strain. *C. albicans* ESCRT (*ARG4*+, *URA3*+, *his1-)* mutants were produced by transposon insertion [28]. Strains were grown in yeast extract-peptone dextrose (YPD) at 30 °C with shaking (250 rpm). For *C. albicans* mutant strains, YPD was supplemented with 80 µg/mL uridine.

### 2.3. Confocal Microscopy

**Figure 1:**
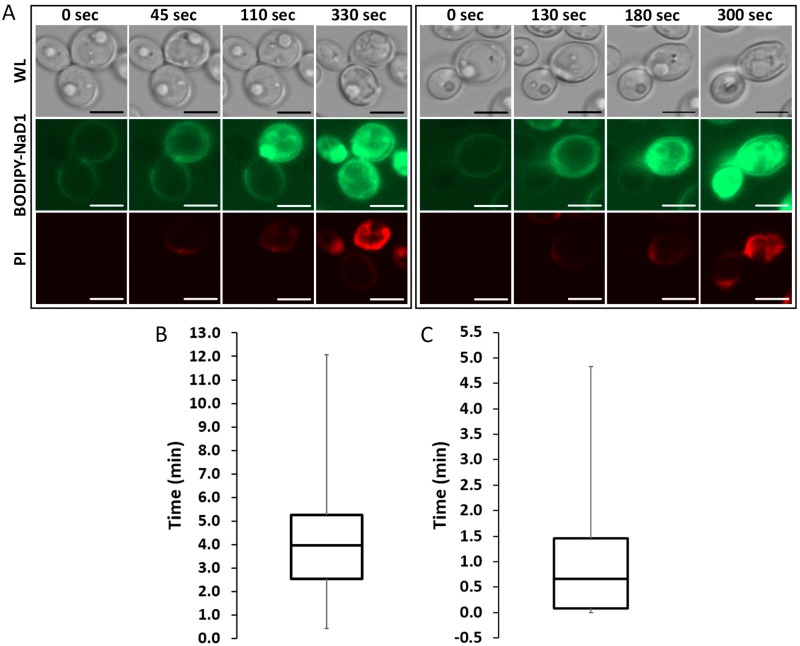
BODIPY-NaD1 uptake occurs in three steps. *C. albicans* DAY185 was treated with 10 µM BODIPY-NaD1 and 5 µM PI and imaged every 5 s with a confocal microscope. (**A**) Each panel shows a *C. albicans* cell at four time-points. Each panel shows the first instance of the described phenotype. White light, BODIPY-NaD1 and PI images are shown for each time point. At the first time-point BODIPY-NaD1 is present on the cell surface. The second time-point shows the moment that BODIPY-NaD1 and PI enter the cytoplasm. By the third time-point the BODIPY-NaD1 has filled the entire cytoplasm. At the last time-point PI accumulation levels off. Scale bars = 5 µM. Images are a representative example of three independent experiments, which gave equivalent results. Cells shown were chosen at random; (**B**) A box-and-whisker plot showing the time delay (in min) from the moment BODIPY-NaD1 is visible on the cell surface to when it first enters the cytoplasm for 70 individual cells. Three independent experiments were performed and a single field of view was analysed for each biological replicate. All cells that were applicable (i.e., had not yet accumulated NaD1 on the cell surface) were analysed and the frames counted between when BODIPY-NaD1 was first observed bound to the cell surface and when uptake occurred; (**C**) A box-and-whisker plot showing the time delay (in min) from the moment BODIPY-NaD1 first enters the cytoplasm to the moment the entire cytoplasm is fluorescent for 95 individual cells. Three independent experiments were performed and a single field of view was analysed for each biological replicate. All cells that were applicable (i.e., had not yet internalised NaD1) were analysed and the frames counted between when uptake first occurred to when NaD1 filled the entire cytoplasm.

*C. albicans* DAY185 overnight cultures were diluted in half-strength potato dextrose broth (PDB; Becton Dickinson, Scoresby, Australia) to an OD_600_ of 0.2 and grown for a further three hours (30 °C, 250 rpm). Cells were then diluted to an OD_600_ of 0.3 with half-strength PDB. For time course experiments, 300 µL aliquots (in 1.5 mL microcentrifuge tubes) were pre-treated with 5 µM propidium iodide (PI; Sigma, St Louis, MO, USA) for 10 min prior to addition of BODIPY- labelled NaD1. For CCCP and latrunculin A experiments, yeast cells were pre-treated with 50 µM CCCP (Sigma) or 100 µM latrunculin A (AdipoGen, Liestal, Switzerland) for 2 h (30 °C, 250 rpm) before addition of 5 µM PI. BODIPY-NaD1 (10 µM) was then added and cells were monitored using a Zeiss LSM510/ConfoCor confocal with images taken every 5 sec. BODIPY was excited at 488 nm (Argon laser) and emission was detected at 505 to 530 nm. PI was excited at 561 nm (DPSS laser) and fluorescence was monitored at 575 to 615 nm. Images were captured using Zen2009 (Zeiss, Oberkochen, Germany) software and analysed using FIJI (Bethesda, Rockville, MD, USA) [29]. Brightness and contrast for Figure 1, Figure 3 and Figure 4 were adjusted using the auto-brightness/contrast function of FIJI.

### 2.4. Flow Cytometry

*C. albicans* DAY185 overnight cultures were diluted in half-strength potato dextrose broth (PDB) to an OD_600_ of 0.2 and grown for 3 h (30 °C, 250 rpm) before they were diluted again to an OD_600_ of 0.1 with half-strength PDB and used in cell death and NaD1 uptake assays. Cell death assays were conducted by treating 300 µL of *C. albicans* cells with 0, 5, 10 or 20 µM of native NaD1 or LL-37 and 5 µM PI. NaD1 uptake at 4 °C was measured by treating 300 µL of cells with 0, 5 or 10 µM BODIPY-labelled NaD1. All treatments were conducted for 30 min at either 30 °C or 4 °C without shaking and in the dark. For brefeldin A and nocodazole experiments, 300 µL of cells were pre-incubated with 40 µM of each inhibitor for 2 h at 30 °C (with shaking). Cells were then treated with 10 µM of BODIPY-NaD1 for 15 min. For all experiments, cells were pelleted after treatment by centrifugation at 13,000 rpm for 2 min. The supernatant was removed and cells were resuspended in 300 µL of 1× phosphate buffered saline (PBS) before analysis using a BD FACSCanto II cytometer (Scoresby, Australia). For BODIPY-labelled NaD1, cells were excited at 488 nm and emission was detected using a 530/30 filter. For PI, cells were excited at 488 nm and emission was detected using a 670LP filter. Data were analysed using Weasel v3.0 (Walter and Eliza Hall Institute, Parkville, Australia).

### 2.5. Growth Inhibition Assays

Fungal growth inhibition assays were performed essentially as described in [30]. Overnight cultures of *C. albicans* cells were diluted to 5000 cells/mL with half-strength PDB. For growth inhibition assays in the presence of latrunculin A, 80 µL of diluted *C. albicans* DAY185 cells were added to 10 µL of latrunculin A and 10 µL of NaD1 in a 96 well microtiter plate (Greiner, Kremsmünster, Austria) to final concentrations of 2.5, 3, 4 and 4.5 µM NaD1 and 0 or 20 μM latrunculin A (AdipoGen). For brefeldin A and nocodazole assays, 80 µL of diluted *C. albicans* DAY185 cells was added to 10 µL of inhibitor and 10 µL of NaD1 in a microtiter plate. Brefeldin A (Sigma) and nocodazole where serially diluted (two-fold) down the plate with top final concentrations of 40 µM and 20 µM respectively. NaD1 was added to all wells at a final concentration of 2.5 µM. No-inhibitor controls were also included. For testing the antifungal activity of NaD1 against the *C. albicans* ESCRT mutants, 80 µL of diluted cells (DAY286 wild type and mutants) were added to 20 µL of NaD1 serially diluted from a top final concentration of 10 µM. All plates were incubated at 30 °C overnight without shaking. Growth of cells was monitored by measuring absorbance at 595 nm in a SpectraMAX M5e plate reader (Molecular Devices, San Jose, CA, USA). Measurements were taken at *t* = 0 and *t* = 24 h.

## 3. Results

### 3.1. NaD1 Uptake into C. albicans Cells Occurs in Three Stages

We have previously described the three step mechanism of action of NaD1 against fungi, which involves binding to the cell surface, internalisation of the peptide into the cytoplasm and induction of reactive oxygen species (ROS) [12,13]. To further investigate the timing of these steps, confocal microscopy time-course experiments were performed using fluorescently labelled NaD1 and the death marker PI. A time-course of the uptake of BODIPY-NaD1 into two individual cells is shown (Figure 1A). In the first cell (left panel), BODIPY-NaD1 is bound to the cell surface and there is no visible PI fluorescence. At 45 sec, BODIPY-NaD1 had started to enter the cytoplasm and a small amount of PI fluorescence was observed. The appearance of the cell did not change for an additional 65 s (110 s time-point), at which time the BODIPY-NaD1 had filled the cytoplasm. Over the following time period, PI fluorescence increased until it levelled off at 330 sec. A similar sequence of events occurred with the second cell (right panel). The median delay between accumulation of NaD1 on the cell surface and uptake into the cytoplasm was 4 min (Figure 1B). The time between the first appearance of NaD1 in the cytoplasm and peak fluorescence in the cytoplasm had a median of 0.67 min (Figure 1C).

### 3.2. NaD1’s Antifungal Activity and Uptake into Fungal Cells is Reduced at 4 °C and after Treatment with CCCP

**Figure 2:**
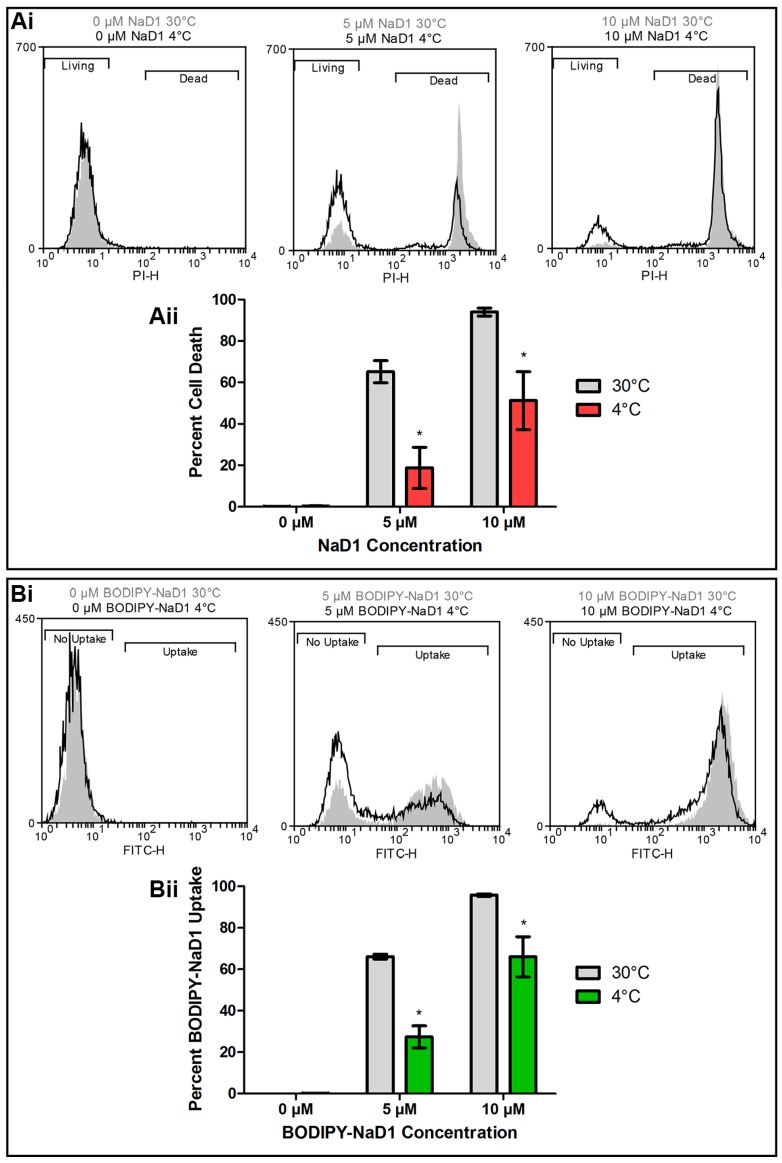
NaD1 induced cell death and uptake is reduced at 4 °C. (**Ai**) *C. albicans* DAY185 was treated with 0, 5 or 10 µM NaD1 for 30 min at 30° and 4 °C. PI (5 µM) was added for quantitation of cell death. Cells with PI fluorescence were counted using a flow cytometer to separate living and dead cells at both 30 °C (grey shading) and 4 °C (black line). Data is a representative example from three independent experiments; (**Aii**) The percentage of PI positive cells (percent cell death) relative to total cell counts was also calculated for three biological replicates and is shown in a bar graph. Error bars are standard error of the means. Values marked by an asterisk are significant (using an independent *t*-test) compared to the 30 °C control (*p*-value < 0.05); (**Bi**) *C. albicans* DAY185 was treated with 0, 5 or 10 µM BODIPY-NaD1 for 30 min at 30° and 4 °C. Cells with BODIPY fluorescence were counted using a flow cytometer and separated to determine the relative numbers of cells that had or had not taken up BODIPY-NaD1 at 30 °C (grey shading) or 4 °C (black line). Data is a representative example from three independent experiments; (**Bii**) The percentage of BODIPY-NaD1 positive cells relative to total cells counts was also calculated for three biological replicates and is shown in a bar graph. Error bars are standard error of the mean. Values marked by an asterisk are significant (using an independent *t*-test) compared to the 30 °C control (*p*-value < 0.05).

At 4 °C, endocytosis and other active cellular processes are blocked [31,32]. To determine if uptake of NaD1 into fungal cells is an energy dependent process, *C. albicans* DAY185 cells were treated with native NaD1 (with PI to monitor cell death), or with BODIPY-labelled NaD1 at 30 °C or 4 °C and uptake into yeast cells was monitored by flow cytometry. There was less cell death when cells were treated with NaD1 for 30 min at 4 °C compared to 30 °C (Figure 2Ai,Aii). In contrast, cell death following treatment with the human cathelicidin LL-37 was not affected by the reduced temperature (Figure S1). To determine if the reduction in cell death at 4 °C had arisen from a decrease in NaD1 uptake into the cytoplasm, the flow cytometry experiment was repeated with fluorescently labelled NaD1. Fewer cells contained BODIPY-labelled NaD1 in the cytoplasm after incubation at 4 °C compared to 30 °C (Figure 2Bi,Bii). This points towards an energy dependent method of uptake, such as endocytosis, as the mechanism by which NaD1 enters *C. albicans* cells.

**Figure 3:**
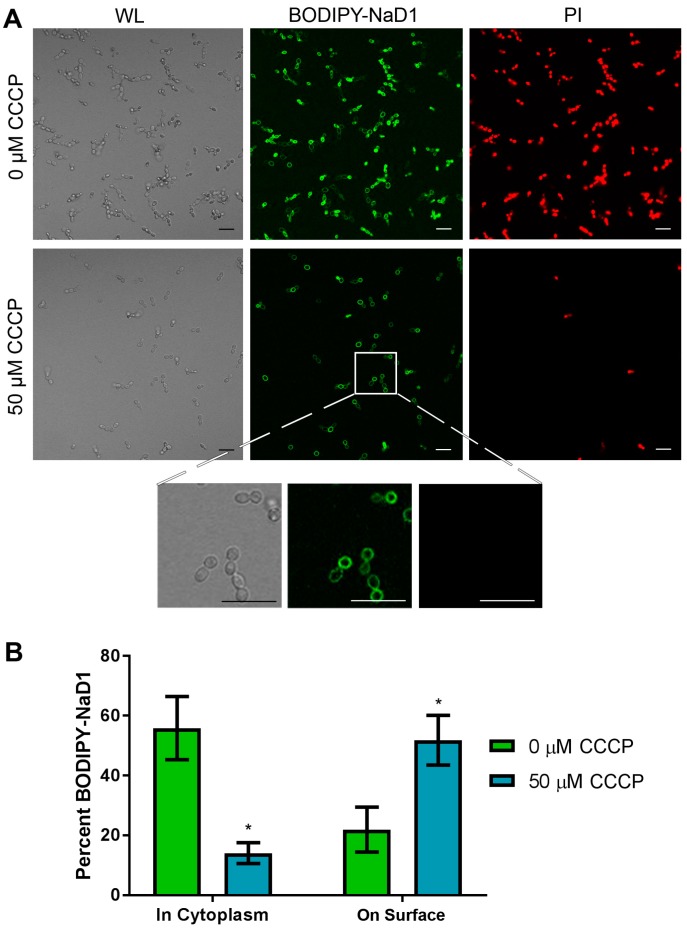
NaD1 uptake is reduced after treatment with CCCP. (**A**) Confocal microscopy was used to monitor uptake of BODIPY-NaD1 into *C. albicans* DAY185 after pre-treatment with CCCP. CCCP pre-treated cells (50 µM CCCP) and no-inhibitor control (0 µM CCCP) cells were treated with 10 µM BODIPY-NaD1 for 10 min. BODIPY-NaD1 and PI and white light images are shown. Scale bars = 40 µm. A subset of cells is also shown with increased magnification. Scale bars = 40 µm. Images are a representative example of three independent experiments, which gave equivalent results. (**B**) Cell counts were performed for four independent experiments and the percentage of BODIPY-labelled NaD1 bound to the membrane or in the cytoplasm was plotted relative to total cell counts. Error bars are standard error of the mean (*n* = 4). Values marked by an asterisk are significant (using an independent *t*-test) compared to the 0 µM CCCP control (*p*-value < 0.05).

An inhibitor of oxidative phosphorylation, CCCP, was used to confirm that the uptake mechanism of NaD1 is energy dependent. As expected, there was a decrease in BODIPY-labelled NaD1 uptake and cell death (monitored by PI fluorescence) when cells were rendered energy deficient with a 50 µM CCCP pre-treatment (Figure 3A). Interestingly, CCCP treatment also increased the proportion of cells with BODIPY-labelled NaD1 on the cell surface. In the absence of CCCP, about 55% of cells had internalised NaD1 and about 21% of cells had accumulated BODIPY-labelled NaD1 on the cell surface. In contrast, about 20% of the CCCP pre-treated cells had NaD1 in the cytoplasm and about 50% of cells had NaD1 restricted to the cell surface (Figure 3B). These data indicate that cell surface binding is not affected by CCCP pre-treatment. However, passage through the plasma membrane requires energy, as evidenced by the reduction in NaD1 uptake and the resulting increase in the number of cells with NaD1 trapped at the cell surface.

### 3.3. NaD1 Uptake and NaD1 Induced Cell Death are Reduced in the Presence of the Actin Assembly Inhibitor Latrunculin A

**Figure 4:**
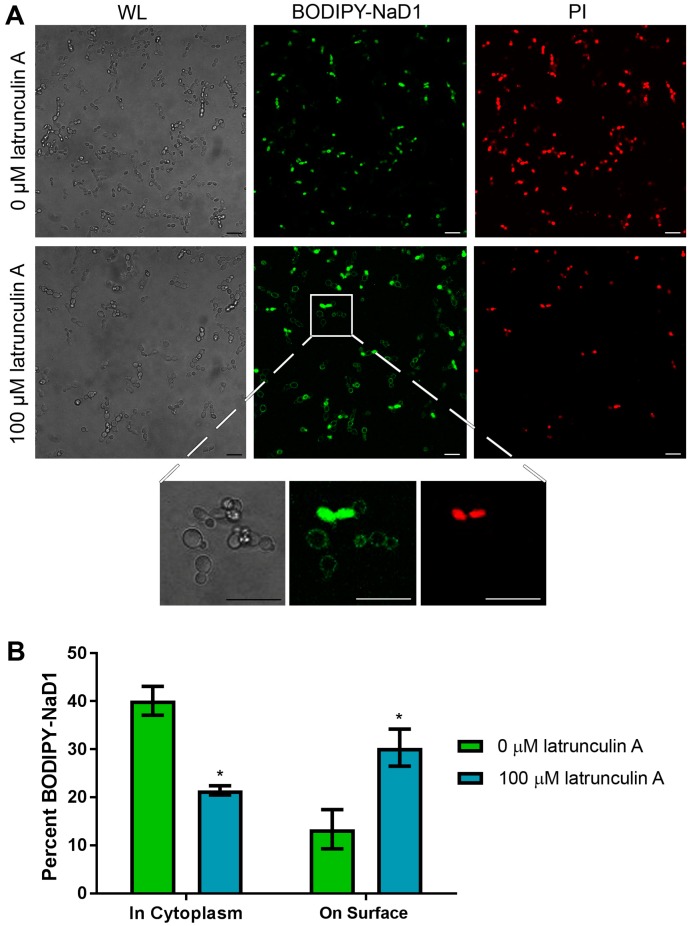
NaD1 uptake is reduced after treatment with latrunculin A. (**A**) Confocal microscopy was used to monitor uptake of BODIPY-NaD1 into *C. albicans* DAY185 after pre-treatment with latrunculin A. Latrunculin A pre-treated cells (100 µM latrunculin A) and no-inhibitor control (0 µM latrunculin A) cells were treated with 10 µM BODIPY-NaD1 for 10 min. BODIPY-NaD1 and PI and white light images are shown. A subset of cells is also shown with increased magnification. Scale bars = 40 µm. Images are a representative example of three independent experiments, which gave equivalent results; (**B**) Cells counts were performed for three independent microscopy experiments and percent BODIPY-labelled NaD1 bound to the membrane or in the cytoplasm was plotted relative to total cell counts. Error bars are standard error of the mean (*n* = 3). Values marked by an asterisk are significant (using an independent *t*-test) compared to the 0 µM latrunculin A control (*p*-value < 0.05).

Having confirmed that uptake of NaD1 into *C. albicans* cells is an energy dependent process, we questioned whether this uptake occurs via endocytosis. This was achieved by pre-treating *C. albicans* cells with the inhibitor latrunculin A which blocks actin polymerisation into filaments, a step which is essential for endocytosis in yeast [19,20]. After *C. albicans* cells were pre-treated for 2 h with 100 µM latrunculin A, uptake of BODIPY-labelled NaD1 was significantly reduced, with approximately 20% of cells containing NaD1 in the cytoplasm after 10 min of NaD1 treatment compared to about 40% of cells in the no-inhibitor control (Figure 4). As with CCCP pre-treatment, there was an increase in the number of cells with NaD1 bound to the cell surface. NaD1 was still bound to the fungal cell surface but internalisation of the peptide was blocked, leaving a higher proportion of cells with surface bound NaD1.

**Figure 5:**
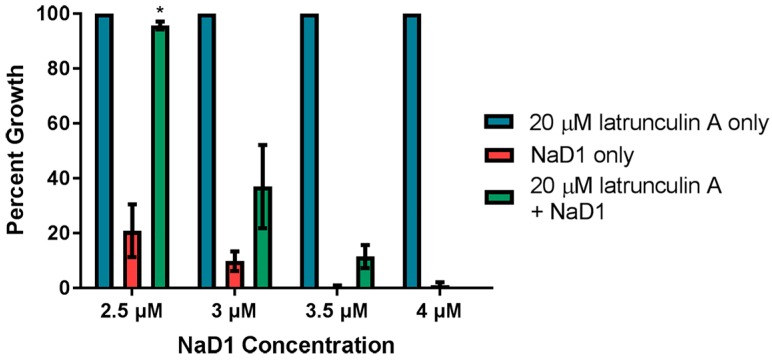
Latrunculin A reduces the inhibitory activity of NaD1 against *C. albicans*. *C. albicans* DAY185 was treated with 20 µM latrunculin A only, NaD1 only or 20 µM latrunculin A and NaD1 in combination. NaD1 was included at 2.5, 3, 3.5 and 4 µM. Data are relative to the 20 µM latrunculin A only control. Error bars represent standard error of the mean (*n* = 3). Values marked by an asterisk are significantly different (using an independent *t*-test) to the NaD1 only control (*p*-value < 0.05).

We also investigated whether latrunculin A blocked uptake when NaD1 was more concentrated. In this assay, *C. albicans* cells were treated concurrently with 20 µM latrunculin A and increasing amounts of NaD1. Lower concentrations of NaD1 were tested in this assay to account for lower starting cell densities used in the fungal inhibition assays. We have previously published data showing that increased cell densities require increased NaD1 concentrations to have a similar level of activity [13]. Growth inhibition by 2.5 µM NaD1 (which is close to the IC_50_ value of 2.3 ± 0.6 µM against *C. albicans* DAY185 previously reported [13]) was reduced in the presence of 20 µM latrunculin A (Figure 5). However, latrunculin A did not protect the *C. albicans* cells when the NaD1 concentration was increased to 4 µM. Thus, endocytosis appears to be the dominant mechanism of NaD1 uptake at lower concentrations but uptake via endocytosis is not be required for cell death at higher NaD1 concentrations (concentrations higher than the IC_50_ value).

### 3.4. Internal Protein Transport Is not Required for NaD1 Activity

**Figure 6:**
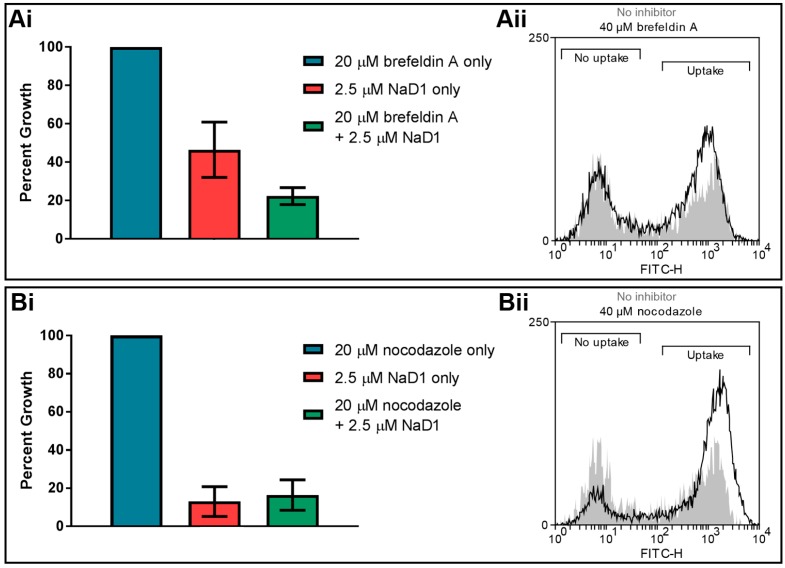
Brefeldin A and nocodazole do not affect the activity of NaD1 against *C. albicans. C. albicans* DAY185 was treated with 2.5 µM NaD1 only, inhibitors only or 2.5 µM NaD1 and (**Ai**) 20 µM brefeldin A or (**Bi**) 20 µM nocodazole in combination. Data are relative to the inhibitor only controls. Error bars represent SEM (*n* = 3). No values were significantly different (using an independent *t*-test) to the NaD1 only control (*p*-value > 0.05). *C. albicans* DAY185 was treated with 10 µM BODIPY-NaD1 for 15 min with (**Aii**) 40 µM brefeldin A or (**Bii**) 40 µM nocodazole. Cells with BODIPY fluorescence were counted using a flow cytometer and separated to determine the percentage of cells that had taken up the BODIPY-NaD1 in the presence (black line) and absence of inhibitors (grey shading).

Once extracellular molecules are internalised by endocytosis, they are transported intracellularly via endosomes. Two inhibitors of endosomal transport were tested in fungal inhibition assays to determine whether endosomal trafficking is critical for NaD1 antifungal activity. The inhibitor brefeldin A blocks retrograde transport from endosomes to the *trans*-Golgi network [33,34], while nocodazole causes depolarization of microtubules leading to defects in endosome movement [35,36]. No significant difference was observed in the growth of *C. albicans* DAY185 cells when they were treated with these two inhibitors (Figure 6). Likewise, deletion of components of the ESCRT pathway, which is responsible for endosome trafficking and formation of late endosomes (or MVBs) [37,38], did not reduce NaD1’s antifungal activity compared to *C. albicans* DAY286 wild type (Table 1). This indicates that retrograde transport is not essential for the activity of NaD1.

## 4. Discussion

Plant defensins are a large family of peptides but the mechanism of action of only a few have been elucidated. Those with identified mechanisms can be divided into two categories, those that enter fungal cells and those that do not. To date, the peptides PsD1, MtDef4, HsAFP1 and NaD1 are the only plant defensins that have been reported to be internalised into cells as part of their mechanism of action [12,39,40,41]. NaD1 traverses the cell wall and plasma membrane of *F. oxysporum f.sp. vasinfectum* and *C. albicans* and accumulates in the cytoplasm [12,13]. In this study, we used time-course experiments with fluorescently labelled NaD1 to investigate the timing of NaD1 uptake into *C. albicans* cells (Figure 1). NaD1 initially accumulated on the cell surface and remained there for a median time of 4 min (Figure 1B). The first stage of uptake then occurred with an influx of NaD1 together with a small amount of PI, into the periphery of the cytoplasm. After another 0.67 min, the NaD1 was distributed across the entire cytoplasm and the organelles had started to disintegrate (Figure 1C). The time delay between attachment of NaD1 to the cell surface and the presence of large amounts of NaD1 and PI in the cytoplasm may reflect an initial uptake of a relatively small amount of NaD1. This relatively small amount of NaD1 may then exert its toxic activity on cytoplasmic targets leading to cell death and membrane permeabilisation and associated influx of NaD1 together with PI. The variability in uptake timing may be due to a threshold amount of protein required to bind the cell surface before uptake of the peptide, or membrane permeabilisation, can occur. It is also possible that cell wall composition changes during the cell cycle may affect the rate of movement of NaD1 through the cell wall. The variability in the timing of NaD1 spread throughout the cytoplasm could indicate that the mechanism of action is complex and requires many stages of action that could be subject to delay.

The interesting observations about the timing of NaD1’s uptake into fungal cells, led to the question of how NaD1 passes through the plasma membrane. Firstly, dependence on energy for uptake of the peptide and cell killing was investigated using the uncoupler of oxidative phosphorylation, CCCP. Treatment with CCCP can block endocytic uptake but also disrupts polyamine transporters, as evidenced by a reduction in uptake of the polyamine spermidine after treatment with CCCP [21]. CCCP also reduces activity and blocks uptake of the human antifungal peptide histatin 5. Mitochondrial petite mutants are also resistant to histatin 5, confirming the energy dependence of histatin 5 activity [42,43]. Two distinct mechanisms of uptake have been observed for this peptide. The first involves translocation of histatin 5 to the cytoplasm by the Dur3p and Dur31p polyamine transporters in *C. albicans* [21,42]. The second involves endocytic uptake and sequestration into the vacuole, with cells eventually undergoing vacuolar expansion and cell death. However, as deletion of genes important for endocytosis or treatment with latrunculin A did not make the cells more sensitive to histatin 5, vacuolar expansion was considered a secondary effect that does not contribute to toxicity [42]. This led to the hypothesis that CCCP induced resistance is due to the blockage of energy dependent uptake through polyamine transporters. In this study, we discovered that uptake of fluorescently labelled NaD1 into *C. albicans* cells was also reduced after addition of CCCP (Figure 3). This supported our earlier work showing that NaD1 activity is reduced against *S. cerevisiae rho* (petite) mutants with reduced mitochondrial function [13]. Combined, this data indicates that uptake of NaD1 is an energy dependent process, such as endocytosis or movement through a polyamine transporter and is not due to passive transport across the membrane.

Polyamine transporters have been tested for a potential role in uptake of NaD1 into *S. cerevisiae* cells [44]. Deletion of the polyamine transport regulator Agp2, which regulates the expression of many genes, including the polyamine transporters Dur3 and Sam3 [45], reduced the antifungal activity and uptake of NaD1 and reduced the activity of the antifungal peptides hβD2, CP29, BMAP-28 and Bac2a [44]. Furthermore, addition of the polyamine, spermidine, protected *S. cerevisiae* against NaD1 induced membrane permeabilisation [44]. However, deletion of the polyamine transporters themselves (*sam3∆, dur3∆* and *sam3∆dur3∆*) did not affect NaD1 activity. Thus it is not likely that NaD1 and the other cationic peptides are internalised by a polyamine transporter, rather the reduction in spermidine uptake leads to an accumulation of positively charged polyamines on the surface of yeast cells, which in turn blocks access of positively charged antimicrobial peptides [44]. Given that NaD1 had bound to the surface of the CCCP treated cells, it is unlikely that the decrease in the activity of NaD1 was due to an accumulation of surface positive charges in these cells. Instead, the decrease in cell killing is likely to be directly linked to the reduction in NaD1 uptake and supports the hypothesis that internalisation of NaD1 into the fungal cytoplasm is an energy dependent process.

To confirm endocytosis as the mechanism of NaD1 uptake in *C. albicans*, cells were treated with NaD1 in the presence of the endocytosis inhibitor latrunculin A, or at 4 °C which reduces endocytic uptake. Lowering the treatment temperature to 4 °C did decrease NaD1 induced cell death and reduced uptake of fluorescently labelled NaD1 into the cytoplasm of *C. albicans* cells (Figure 2). In contrast, cell death induced by the human antimicrobial peptide LL-37 was not reduced when the temperature was lowered to 4 °C (Figure S1) This is consistent with a report by Ordonez and co-workers (2014), in which they suggest that LL-37 killing arises from direct membrane permeabilisation and does not involve endocytosis, because treatment with azide to reduce energy production failed to inhibit LL-37 activity [46,47]. Similar to NaD1, the *Penicillium* antimicrobial peptide PAF also appears to be internalised by endocytosis, because latrunculin B, which inhibits actin polymerisation like latrunculin A, blocked PAF internalisation into *Aspergillus nidulans* hyphae [18]. In addition, PAF like NaD1 did not enter cells in the presence of CCCP and PAF uptake was blocked at 4 °C [18].

In another scenario, NaD1 may not be directly internalised by endocytosis but a target of NaD1 on the plasma membrane may require turnover or regulation by endocytosis. This occurs with the ether-phospholipid edelfosine in *S. cerevisiae*. Edelfosine is internalised into *S. cerevisiae* cells but not through endocytosis and its uptake is not required for toxicity. Instead, edelfosine functions by inducing endocytic uptake of the plasma membrane H^+^ pump Pma1p, which is transported to the vacuole for degradation. Endocytosis mutants were less susceptible to edelfosine than wild type cells, even though edelfosine uptake was not affected in these mutants. Instead, blocking endocytosis had reduced antifungal activity by stopping internalisation of Pma1p [48]. In addition, mutants with deletions of genes encoding proteins required for protein recycling and transport, including ESCRT complexes, also enhanced resistance to edelfosine. Uptake and transport of edelfosine to the ER in these mutants was also not altered and resistance in these mutants was attributed to higher levels of Pma1p in the membrane due to decreased protein turnover [48]. As mutants in the ESCRT pathway were not resistant to NaD1 (Table 1) it is unlikely that an unknown target protein in the membrane is responsible for the NaD1 resistance that is generated by treatment with latrunculin A.

Endocytosis may not be the only mechanism by which NaD1 enters into cells. Latrunculin A did not protect *C. albicans* from the deleterious effects of NaD1 when NaD1 was at concentrations higher than 3.5 µM in a fungal inhibition assay (Figure 5). However, endocytosis may not have been completely blocked in cells treated with latrunculin A and a limited amount of endocytosis at high NaD1 concentrations may have been sufficient to internalise a lethal concentration of NaD1.

Alternatively, NaD1 at high concentrations may have directly damaged the plasma membrane leading to cell death or transient lipid bilayer disruption which allowed entry of some NaD1 into the cytoplasm. We have previously reported that NaD1 at high concentrations does partially penetrate artificial phosphatidylinositol bilayers. Even though the resulting membrane disorder was reversible, the association of NaD1 with the membrane may have been sufficient to allow small amounts of NaD1 to come into contact with the phosphatidylinositol 4,5 bisphosphate (PI(4,5)P_2_) on the inner leaflet of the membrane. Association of even small amounts of NaD1 with PI(4,5)P_2_ bilayers results in tight irreversible binding that produces severe disorder of the membrane and membrane disruption [16]. A similar mechanism may occur with histatin 5 at high concentrations. That is, energy depleted *C. albicans* cells accumulate high levels of histatin 5 in their cell walls and are killed by histatin 5 via direct interaction with the membrane [42].

Interestingly, El-Mounadi and co-workers [15] have reported that the mechanism of uptake of the plant defensin MtDef4 varies between fungal species. Entry of MtDef4 into *Neurospora crassa* cells is energy dependent and involves endocytosis. Consequently, uptake of MtDef4 into *N. crassa* is abolished when ATP production is inhibited by sodium azide. Internalisation of MtDef4 is also reduced by the endocytosis inhibitor filipin, which blocks lipid-raft mediated endocytosis [15]. In contrast, MtDef4 uptake into *Fusarium graminearum* is only partially blocked by sodium azide and none of the tested endocytosis inhibitors affected internalisation of the defensin. Instead, MtDef4 is hypothesised to be internalised by translocation using a partially energy-dependent mechanism [15]. Therefore, it is possible that uptake of NaD1 could occur through a different mechanism in different fungal species. It is necessary to expand research into further species to confirm this.

Interestingly, treatment with latrunculin A did not affect MtDef4 uptake into *N. crassa.* In addition, uptake ofMtDef4 was affected by brefeldin A [15]. These results are in direct contrast to observations with NaD1 and indicate that these peptides are internalised by different forms of endocytosis. Unlike MDef4, NaD1 movement through the cell does not appear dependent on the internal endosomal system, also evidenced by the failure of nocodazole and the deletion of ESCRT components to alter sensitivity to NaD1 (Figure 6, Table 1). This is consistent with the findings that while MtDef4 most likely targets the vacuole of *N. crassa* hyphae [15], NaD1 does not appear to associate with any particular organelle, instead diffusing through the entire cytoplasm [12]. It is not known how NaD1 escapes the endocytic vesicles to access the cytoplasm.

Nonetheless, these observations further expand the model of NaD1’s mechanism of action. NaD1 traverses the cell wall, accumulates on the cell surface and is then endocytosed through the plasma membrane into the cytoplasm where it kills the fungal cell, possibly through production of reactive oxygen species and by membrane disruption following binding of NaD1 to PI(4,5)P_2_ on the inner leaflet.

## Figures and Tables

**Table 1:** Deletion of ESCRT pathway components does not affect activity of NaD1.

IC_50_ (µM)							
Wild type (DAY286)	*vps2Δ*	*vps23Δ*	*vps24Δ*	*vps28Δ*	*vps36Δ*	*snf7Δ*	*bro1Δ*
1.8 ± 0.49	1.8 ± 0.40	1.8 ± 0.43	2.1 ± 0.33	1.6 ± 0.33	1.7 ± 0.28	1.9 ± 0.45	2.1 ± 0.35

*C. albicans* DAY286 (WT), *vps2Δ*, *vps23Δ*, *vps24Δ*, *vps28Δ*, *vps36Δ*, *snf7Δ* and *bro1Δ* were treated with various concentrations of NaD1. IC50 ± standard deviation of *C. albicans* ESCRT mutants treated with NaD1 are shown (*n* = 4). No values were significantly (using an independent *t*-test) different from wild-type (*p* > 0.05).

## Supplementary Materials

The following are available online at http://www.mdpi.com/2309-608X/4/1/20/s1, Figure S1: LL-37 induced cell death was not reduced when the temperature is lowered from 30 °C to 4 °C.
