# Supplementary material for: The Plant Defensin NaD1 Enters the Cytoplasm of Candida albicans via Endocytosis
Source: J Fungi (Basel). 2018 Feb 6;4(1):20. doi: 10.3390/jof4010020 (PMC5872323; doi:10.3390/jof4010020)
Supplement: Supplementary file 1 [file jof-04-00020-s001.pdf]

The Plant Defensin NaD1 Enters the Cytoplasm of *Candida Albicans* via Endocytosis

Brigitte M. E. Hayes, Mark R. Bleackley, Marilyn A. Anderson and Nicole L. van der Weerden *

La Trobe Institute for Molecular Science, 3086 Melbourne, Australia; B.Hayes@latrobe.edu.au (B.M.E.H.); m.bleackley@latrobe.edu.au (M.R.B.); M.Anderson@latrobe.edu.au (M.A.A.)

* Correspondence: N.VanDerWeerden@latrobe.edu.au

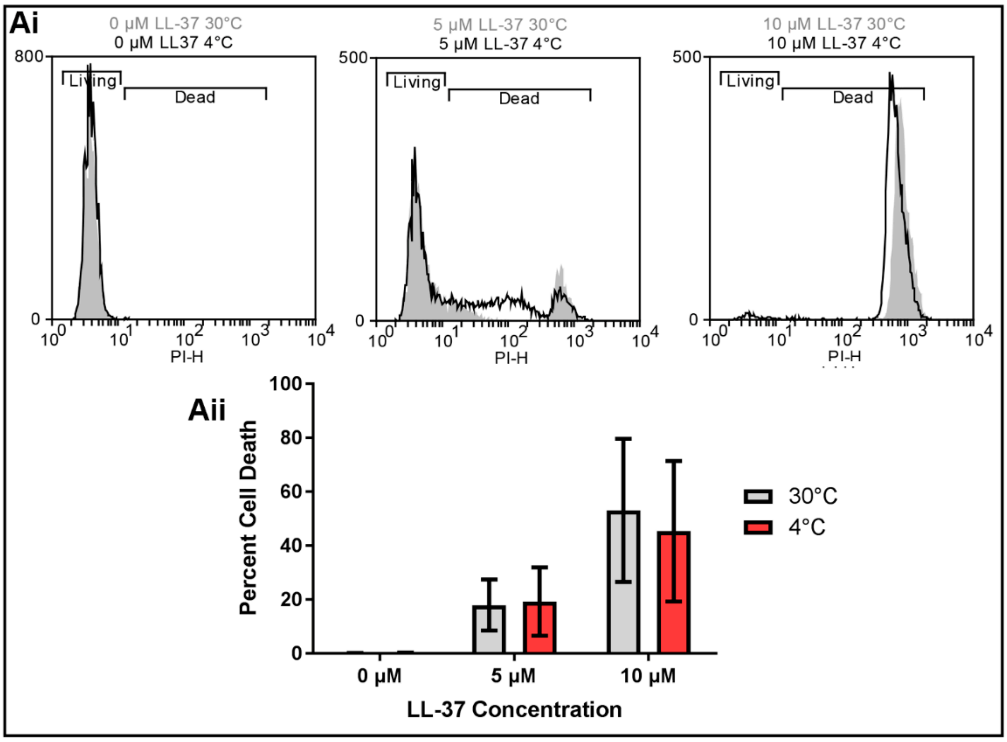

Figure S1. LL-37 induced cell death was not reduced when the temperature is lowered from 30 °C to 4 °C.
